# Nicotine consumption may lead to aseptic loosening in proximal mega-prosthetic femoral replacement

**DOI:** 10.1007/s10195-016-0426-7

**Published:** 2016-08-17

**Authors:** Philip J. F. Leute, Isabel Hoffmann, Ahmed Hammad, Stefan Lakemeier, Hans-Michael Klinger, Mike H. Baums

**Affiliations:** 1 Department of Orthopedic Surgery, University of Göttingen, Robert-Koch-Straße 40, 37075 Göttingen, Germany; 2 Department of Orthopedic Surgery, Helios Aukammklinik, Leibnizstraße 21, 65191 Wiesbaden, Germany

**Keywords:** Smoking, Nicotine, Complication, Femur, Proximal, Replacement, Aseptic, Loosening

## Abstract

**Background:**

Aseptic loosening after total hip arthroplasty is likely related to nicotine ingestion. However, aseptic loosening as a direct consequence of smoking habits has not been described with regard to proximal mega-prosthetic femoral replacement. The aim of the present study was to evaluate the association between nicotine consumption and aseptic loosening rates after proximal mega-prosthetic femoral replacement.

**Materials and methods:**

A consecutive series of patients who received mega-prosthetic replacement of the proximal femur at our hospital between 2005 and 2015 were included. Their files were reviewed and evaluated for the influence of smoking on aseptic loosening rates. All living patients were invited to complete a functional follow-up assessment at our clinic.

**Results:**

Twenty-six patients with 27 prostheses were included. Five patients were active smokers, and 21 patients were non-smokers. Aseptic loosening was observed in three patients in the smoking group, whereas none of the non-smokers developed aseptic loosening. Fisher’s exact test showed a relationship between nicotine consumption and aseptic loosening of the prostheses (*p* = 0.003).

**Conclusions:**

Smoking increases the likelihood of aseptic loosening after proximal mega-prosthetic femoral replacement.

**Level of evidence:**

Level 4 according to Oxford Centre of Evidence-Based Medicine 2011.

**Electronic supplementary material:**

The online version of this article (doi:10.1007/s10195-016-0426-7) contains supplementary material, which is available to authorized users.

## Introduction

**Fig. 1:**
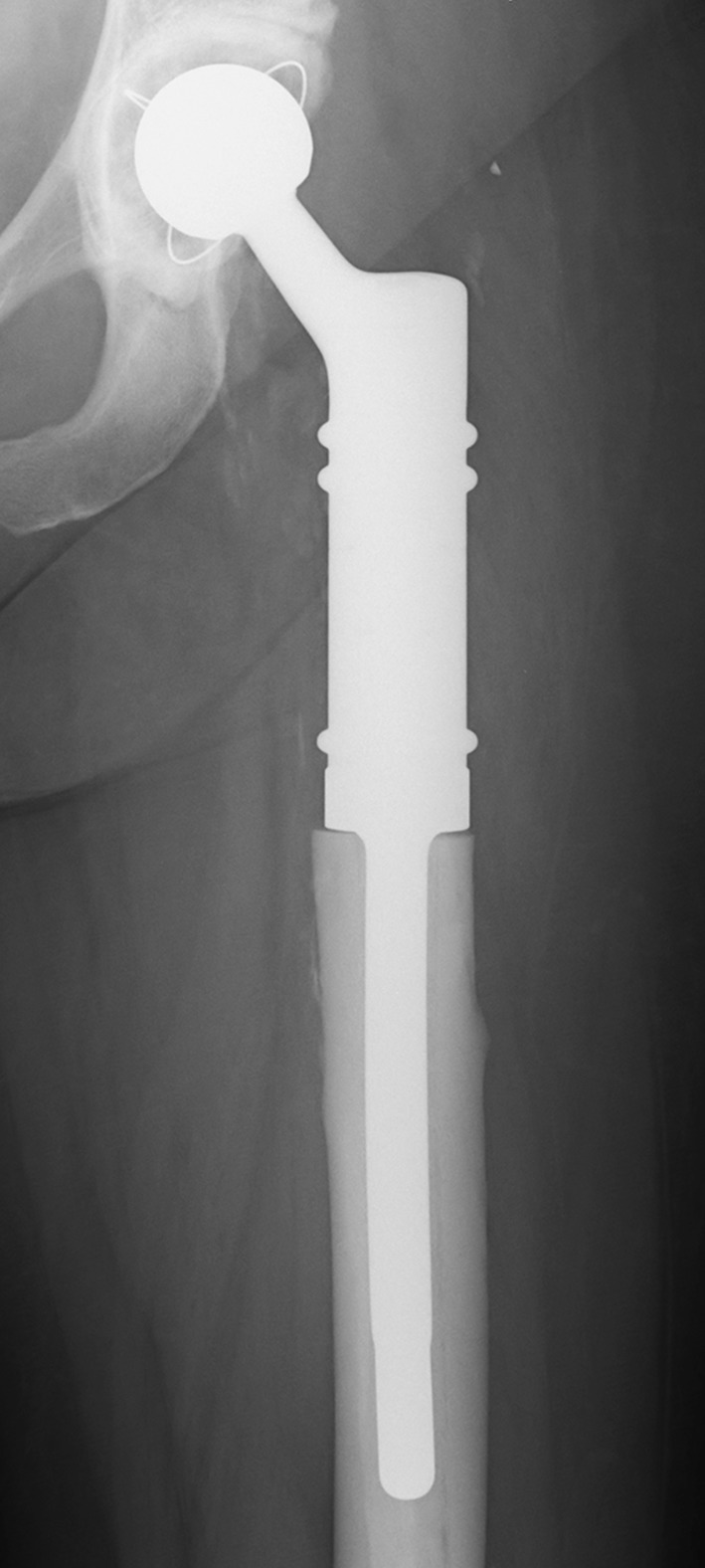
Proximal femur replacement

Mega-prosthetic replacement of the proximal femur has become a standard procedure to treat primary bone tumors, metastases and large bone defects after orthopedic surgeries using a large, special type of total hip arthroplasty (Fig. 1). The orthopedic surgeon must understand the reasons for postoperative complications to avoid them and ensure that the patient has a high-quality postoperative outcome. Although various complications occur after the implantation of these prostheses, aseptic loosening rates are of particular interest because they are notably higher than after standard total hip arthroplasty (5–27 %) [1–3]. A large meta-analysis recently demonstrated that nicotine consumption was associated with higher aseptic loosening rates after standard total hip arthroplasty [4]. However, smoking has not been established as a risk factor for aseptic loosening after mega-prosthetic proximal femoral replacement. Considering the aforementioned recent study results regarding standard total hip arthroplasty, we hypothesized that smoking habits also influence the aseptic loosening rates of mega-prosthetic proximal femoral replacement. Therefore, this study sought to test the correlation between the aseptic loosening of these prostheses and nicotine consumption.

## Materials and methods

### Protocol

In accordance with our unpublished study protocol ‘Nicotine consumption and proximal femoral replacement: a 10-year single-center analysis’, the present study was designed as a retrospective, single-center study with a one-time cross-sectional functional follow-up assessment for all surviving patients. We planned to include at least 20 patients with seven different surgeons in this study. To support our hypothesis, the patients would be segregated into smoking and non-smoking groups, and the aseptic loosening rates would be compared between groups. Smokers were defined as patients who had consumed nicotine before and after implantation of the femoral mega-prosthesis, whereas all other patients were defined as non-smokers. Aseptic loosening was defined based on pain upon ambulation and radiological evidence of periprosthetic bone resorption without signs of infection (via culture-positive swabs or blood samples). Radiological evidence of periprosthetic bone resorption was obtained using standardized X-rays (antero-posterior and Lauenstein’s oblique lateral projection) that were independently evaluated by a radiologist and an orthopedic surgeon. A microbiologist analyzed intraoperatively obtained swabs that were considered positive when the same bacteria were present in two of five swabs or when the patient had additional signs of bacteremia (elevated C-reactive protein [CRP] levels or leukocytosis).

### Statistical analyses

The differences in aseptic loosening rates between the groups of smokers and non-smokers were analyzed using Fisher’s two-tailed exact test because it is reliable for small sample sizes. In addition, a Kaplan–Meier analysis was used to evaluate the patient and prosthesis survival rates. The means, standard deviations (SDs), medians and ranges were calculated for all data. All statistics were computed using StatSoft, Inc. (2014) STATISTICA data analysis software (StatSoft, Inc., Tulsa, OK, USA), version 12.0.

### Methodological approach

To obtain our study group, we requested information from the controlling department of our hospital, where all diagnoses and procedures are matched to the treated patients and retained. All potential cases of proximal mega-prosthetic femoral replacement between 2005 and 2015 were identified according to the International Classification of Procedures in Medicine (ICPM) figures that were used to code operations involving femoral replacement with special prostheses (confer Supplementary Material). All patient files and X-rays were reviewed, and all patients who received special femur prostheses other than a proximal femoral replacement were excluded. Only cases in which the surgical procedure was comparable with that used for these implants were included (i.e., transection of the proximal femur in the subtrochanteric region and replacement of the proximal part using an implant with a large monopolar femoral head and a cone for intramedullary insertion into the femur). Only those cases in which the implants were cemented and not attached to the bone with additional screws or plates were included.

### Patient baseline characteristics

Baseline data including sex, age and preoperative data regarding body mass index (BMI), American Society of Anesthesiologists (ASA) physical status [5], number and type of concomitant diseases, number and type of medications, alcohol consumption and use of illicit drugs were documented based on the available patient files. The surgical protocols were reviewed, and the data regarding the implant manufacturer, operating surgeon, cementation, implants at the surgical site prior to implantation, intraoperative complications and duration of surgery were recorded. The postoperative period was investigated, and data were recorded regarding the application, type and amount of postoperative antibiotics, duration of bed rest, duration of days until fully weight bearing, rehabilitation after inpatient treatment, postoperative chemotherapy or radiation, duration of inpatient treatment and postoperative complications.

### Nicotine consumption and aseptic loosening

Next, the data regarding nicotine consumption prior to surgery were recorded. Smoking habits (i.e., amount and duration in cigarette pack years [py], consumption of tobacco via cigar, pipe or snuff tobacco) prior to surgery were documented based on patient files because the ward nurses routinely record this information for every patient upon admission. To determine postoperative nicotine consumption, the patients (or their living relatives when the patients were deceased) were contacted and interviewed about their smoking habits after proximal femur replacement.

All of the documentation from the patient follow-up assessments and subsequent operations with the attendant X-rays were then reviewed to determine how many patients from each group developed aseptic loosening.

### Functional follow-up assessment

All living patients were invited to complete a functional follow-up assessment at our clinic. These evaluations were performed using the harris hip score (HHS) [6], toronto extremity salvage score (TESS) [7], musculoskeletal tumor society score (MSTS) [8] and short form 36 (SF36) [9]. Moreover, leg length was recorded for every patient and compared with the documentation in their files or measured at the functional follow-up assessment.

## Results

### Methodological approach

A total of 225 cases of special femoral prostheses implantation were identified. All of the patient files and X-rays were reviewed, resulting in the exclusion of 198 patients who had not received a proximal femoral replacement but a different femoral prosthesis or had undergone a surgical procedure that differed from the study protocol.

### Patient baseline characteristics

Twenty-six patients who received proximal femoral replacements were included in this study. Fourteen women and 12 men received 27 prostheses, including one patient who received bilateral prostheses. All protheses stems were cemented with gentamicin-loaded polymethylmethacrylate bone cement. The indications were metastatic bone disease in 16 cases, malignant primary bone tumors in six cases, bone loss after orthopedic surgery in four cases and a benign bone tumor in one case (Table 1).

**Table 1:** Patient baseline characteristics and complications

Patient	Gender	Age	DM	RA	Nicotine	Reason for replacement	Implant at hip prior to operation	Manufacturer
1	M	76.0	–	–	NS	Metastasis urothel-carcinoma	–	Cremascoli
2	M	66.8	–	–	S (49 py)	Malignant histiocytoma	–	Cremascoli
3	F	66.9	–	–	NS	Metastasis mamma-carcinoma	–	Implantcast
4	F	75.4	–	–	NS	Metastasis mamma-carcinoma	–	LINK
5	F	62.3	Yes	–	NS	Metastasis mamma-carcinoma	–	Implantcast
6	M	73.6	–	–	NS	Plasmocytoma	Standard hip endoprosthesis	Implantcast
7	F	66.9	Yes	–	NS	Plasmocytoma	–	Implantcast
8	M	73.3	–	–	S (52 py)	Metastatic adenocarcinoma prostate	–	Implantcast
9	M	37.6	–	–	S (20 py)	Chondrosarcoma	–	Implantcast
10 left leg	F	60.0	–	–	NS	Metastasis mamma-carcinoma	Intramedullary femur nail	Implantcast
10 right leg	F	59.8	–	–	NS	Metastasis mamma-carcinoma	Intramedullary femur nail	Implantcast
11	M	55.4	–	–	S (36 py)	Fibrous dysplasia, pathological fracture	–	Implantcast
12	F	56.6	–	–	NS	Metastasis mamma-carcinoma	Intramedullary femur nail	Eska Implants
13	M	56.2	–	–	NS	Metastatic renal cell carcinoma	–	Eska implants
14	F	77.5	Yes	Yes	NS	Bone loss after osteosynthesis	Intramedullary femur nail	Eska implants
15	F	66.4	–	–	NS	Metastatic renal cell carcinoma	Metal plate	Eska implants
16	M	52.9	–	–	NS	Metastatic renal cell carcinoma	–	Eska implants
17	F	75.7	–	–	NS	Metastatic renal cell carcinoma	–	Eska implants
18	M	81.7	–	–	S (67 py)	Bone loss after removal of hip prosthesis	Standard hip endoprosthesis	Eska Implants
19	M	59.7	–	–	NS	Chondrosarcoma	Standard hip endoprosthesis	Implantcast
20	F	74.0	–	–	NS	Plasmocytoma	Intramedullary femur nail	Eska implants
21	F	66.6	–	–	NS	Metastasis mamma-carcinoma	Intramedullary femur nail	Eska implants
22	F	72.8	–	–	NS	Bone loss after removal of hip prosthesis	Standard hip and knee endoprosthesis	Eska implants
23	M	70.4	–	–	NS	Metastatic renal cell carcinoma	–	Orthodynamics
24	F	57.6	–	–	NS	Bone loss after osteosynthesis	Intramedullary femur nail	Orthodynamics
25	M	77.1	Yes	Yes	NS	Metastasis bronchial adenocarcinoma	–	Orthodynamics
26	F	60.9	–	–	NS	Metastasis mamma-carcinoma	–	Orthodynamics

Patient	Duration of operation (min)	Intra-/postoperative complications	Time primary operation-first following operation	Reasons for all implant related following operations	Amount of following related operations	Time to removal of implant (months)	Situation after implant removal	Time from implantation yo death or alive
1	267	–/Wound infection, lymphatic edema	10.2 months	Superficial infection (enterococcus faecalis, staphylococcus epidermidis)	1			0.9 years
2		–/Hematoma, dislocation <3 months after surgery	1 day	Hematoma, dislocation	2			1.0 years
3	158	–/–						0.4 years
4	146	–/–						0.6 years
5	143	–/–						3.0 years
6	260	–/Dislocation >3 months after surgery						1.3 years
7	108	–/–						1.7 years
8	155	–/–						0.1 years
9	300	–/Aseptic shaft loosening	71.0 months	Aseptic shaft loosening	1	71.0	Re-implantated proximal cemented femoral replacement	Alive
10 left leg		–/Dislocation >3 months after surgery						3.3 years
10 right leg		–/–						3.5 years
11	229	–/Aseptic shaft loosening	12.0 months	Aseptic shaft loosening	1	12.0	Re-implantated proximal cemented femoral replacement	Alive
12	273	–/Wound infection, urinary tract infection	6.0 months	Superficial infection (staphylococcus epidermidis, corynebacterium species)	4			0.8 years
13	242	–/Wound infection	5.2 months	Superficial infection (staphylococcus aureus)	1			0.5 years
14	306	–/Sigma diverticulitis						0.7 years
15	201	–/Wound healing deficiency without infection	4.2 months	Wound healing deficiency without infection	1			0.9 years
16	269	–/Dislocation <3 months after surgery	2.4 months	Dislocation	1			5.3 years
17	219	–/–						Alive
18	341	Femoral vein lesion / aseptic shaft loosening	45.5 months	Aseptic shaft loosening	1	45.5	Total femoral replacement	Alive
19		–/Gluteal insufficiency, local tumor recurrence	82.9 months	Local tumor recurrence	1	82.9	Hip ex-articulation without implant	Alive
20	240	–/Dislocation >3 months after surgery, recurrent	43.3 months	Dislocation	1			Alive
21	244	–/Wound infection, hematoma, urinary tract infection	0.4 months	Superficial infection (staphylococcus haemolyticus), hematoma	2			0.7 years
22	295	–/Wound infection	12.5 months	Deep infection/septic loosening (MRSA, MRSE, staphylococcus capitis, campylobacter fetus)	5	13.9	Girdlestone situation with attendant femoral amputation	Alive
23	240	–/Dislocation >3 months after surgery						Alive
24	220	–/–						Alive
25	183	–/Dislocation >3 months after surgery						0.4 years
26	224	–/–						Alive

*M* male, *F* female, *NS* non-smoker, *S* smoker, *DM* diabetes mellitus, *RA* rheumatoid arthritis, *Py* pack years of nicotine consumption, *MRSA* methicillin-resistant *staphylococcus aureus*, *MRSE* methicillin-resistant *staphylococcus epidermidis*

The average age at surgery was 65.9 years (SD ± 9.9, median 66.8, range 37.6–81.7) with an average BMI of 27.4 (SD ± 5.6, median 26.0, range 16.4–41.1). The patients had 4.4 concomitant diseases on average (SD ± 2.1, median 5.0, range 1.0–7.0) and were prescribed 5.4 medications on average (SD ± 3.5, median 5.0, range 1.0–14.0). The average ASA score at surgery was 2.5 (SD ± 0.6, median 3.0, range 1.0–3.0).

The average duration of surgery was 228.8 min (SD ± 59.2, median 240.0, range 108.0–341.0). Only one of 27 surgeries produced an intraoperative complication (femoral vein lesion). This lesion was sutured immediately and healed without further incident. During the postoperative period, the average duration of bed rest was 6.3 days (SD ± 4.4, median 7.0, range 0.0–14.0), and the average number of days until patients were fully weight bearing was 27.4 (SD ± 23.8, median 16.5, range 0.0–100.0). Twenty-three of 27 patients received postoperative antibiotics (18 received cefuroxime; five received cefuroxime and clindamycin) for an average of 26 days (SD ± 33.7, median 10.0, range 3.0–90.0). Inpatient treatment lasted 31.0 days on average (SD ± 16.5, median 27.0, range 8.0–94.0). Sixteen of 27 patients underwent postoperative rehabilitation. The application of postoperative chemotherapy was documented for 7 of 27 patients, and postoperative radiation was documented for 9 of 27 patients. Three of our patients regularly consumed alcohol, but none of these patients used illicit drugs.

### Nicotine consumption and aseptic loosening

**Fig. 2:**
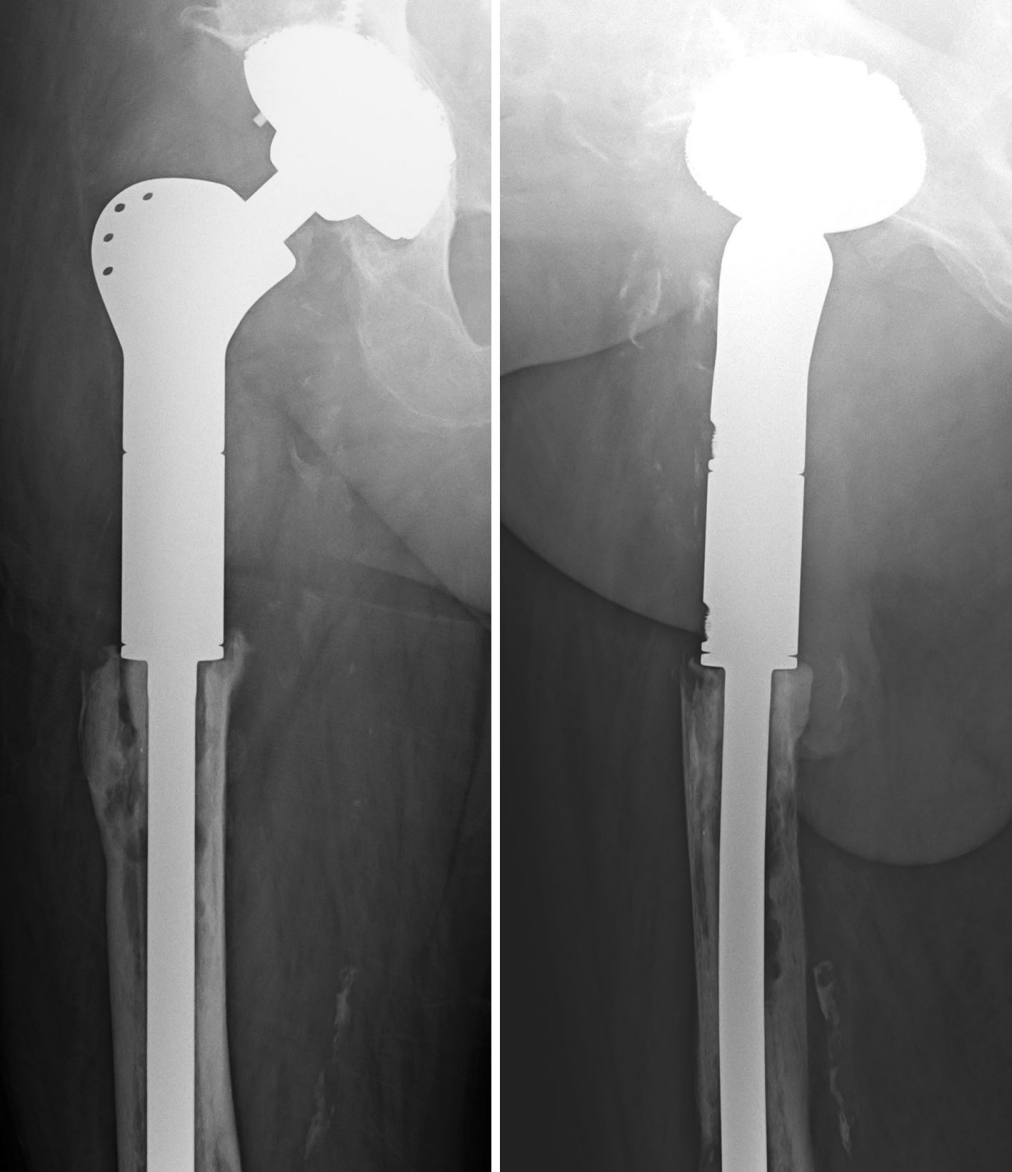
Aseptic loosening

Five patients were smokers, whereas 21 patients were non-smokers. The smokers consumed nicotine via cigarettes prior to implantation for an average of 44.8 py (SD ± 17.7, median 49, range 20–67). None of the patients ingested tobacco via cigar, pipe or snuffing. After an average follow-up period of 2.7 years (SD ± 2.3, median 1.7, range 0.1–7.9), the X-rays revealed signs of bone resorption in four of 27 prostheses (Fig. 2).

One of these patients showed microbiological testing results indicating infection with methicillin-resistant *Staphylococcus aureus* (MRSA) and methicillin-resistant *S. epidermidis* (MRSE) with leukocytosis and elevated CRP levels. Thus, this patient was classified as having septic loosening. This patient underwent a girdlestone procedure with additional above-knee amputation due to concomitant infection of the adjacent knee prosthesis.

The remaining three patients did not show bacterial growth according to microbiological tests and did not have signs of infection in their blood; hence, they were classified as having aseptic loosening. Throughout the course of the follow-up assessments of two of these patients, the proximal femoral replacement was exchanged for a similar proximal mega-prosthesis, whereas the third patient received a total femoral replacement. All three of these patients were smokers, whereas none of the non-smokers developed aseptic loosening. Fisher’s two-tailed exact test revealed a significant relationship between nicotine consumption (smoking) and aseptic loosening (*p* = 0.003).

### Functional follow-up assessment

**Fig. 3:**
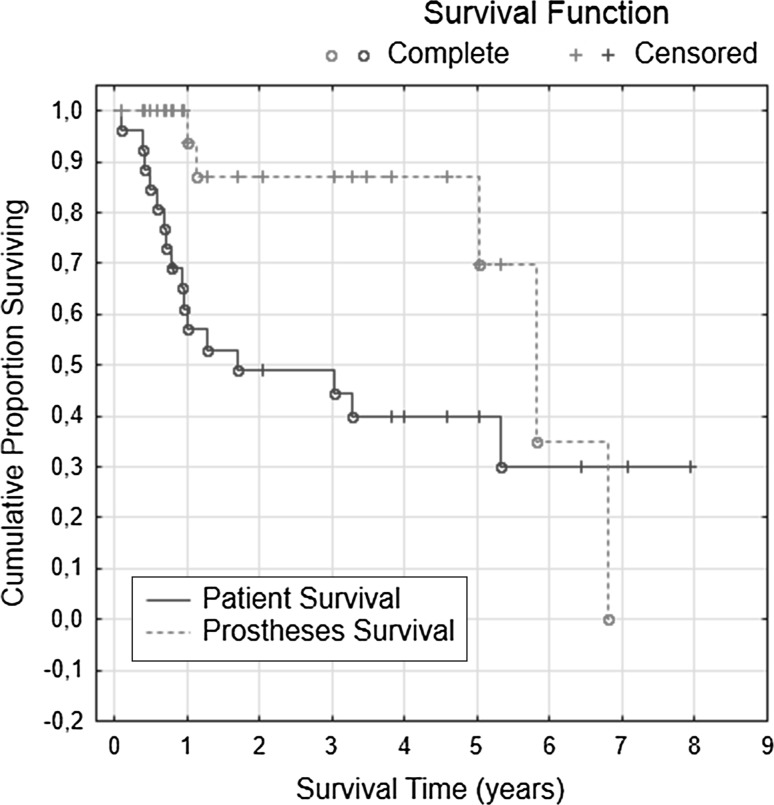
Kaplan−Meier analysis

Ten of 26 patients were alive at the time of this study, with eight of 27 prostheses remaining; all of these patients were available for a functional follow-up assessment (Table 2). No patient death (16/26) was related to the implantation of the mega-prosthesis or to any subsequent complication. The average age at the follow-up assessment was 69.2 years (SD ± 12.5; median 70.9; range 44.6–86.7). The time from primary implantation to the follow-up assessment was 4.7 years on average (SD ± 2.2; median 4.8; range 0.8–7.9). Of the 10 survivors, five had the original implant; however, the implant was removed from the hips of the other five patients. The average time between implantation and removal was 45.1 months (SD ± 32.3; median 45.5; range 12.0–82.9). Apart from the aforementioned three cases of aseptic loosening and one case of septic loosening, one patient underwent hip ex-articulation because of local tumor recurrence (chondrosarcoma). Another patient was wheelchair-bound due to Parkinson’s disease and multiple osteoporotic spinal fractures. A Kaplan–Meier analysis revealed that the prosthesis survival rates were generally higher than the patient survival rates (Fig. 3).

**Table 2:** Functional follow-up

Patient	Age at follow-up (years)	Time from implantation until functional follow-up (years)	Hip situation at follow-up	Knee society score (%)	Harris hip score (%)	Toronto extremity salvage score (%)	Musculoskeletal tumor society score (%)	Musculoskeletal tumor society score (points out of 30)	Short form 36 physical functioning
9	44.6	7.1	Proximal cemented femoral replacement			73.6	50	15	30
11	61.8	6.4	Proximal cemented femoral replacement—wheelchair bound due to Parkinson’s disease and osteoporotic spinal fractures			34	10	3	20
17	80.7	5.0	Proximal cemented femoral replacement		90	85	93.3	28	100
18	86.7	5.0	Total femoral replacement	83	65	57	50	15	25
19	67.6	7.9	Hip ex-articulation			76	40	12	35
20	78.6	4.6	Proximal cemented femoral replacement		51	52.8	33.3	10	15
22	76.8	4.0	Girdlestone situation with femur amputation			48.3	63.3	19	10
23	74.2	3.8	Proximal cemented femoral replacement		72	64.3	56.7	17	25
24	59.6	2.0	Proximal cemented femoral replacement		94	91.1	96.7	29	75
26	61.7	0.8	Proximal cemented femoral replacement		93	68	80	24	50

Patient	Short form 36 role-physical	Short form 36 bodily pain	Short form 36 general health	Short form 36 vitality	Short form 36 social functioning	Short form 36 role-emotional	Short form 36 mental health	Short form 36 physical component summary measure	Short form 36 mental component summary measure
9	100	84	20	30	25	33.3	44	42.9	29.7
11	0	10	20	30	12.5	0	24	25.1	26.1
17	100	100	67	95	100	100	100	53.7	61.8
18	100	84	52	55	87.5	100	68	39.2	55.5
19	0	62	77	75	87.5	100	72	30.6	62.2
20	0	41	20	50	100	0	52	24.9	45.7
22	0	41	67	50	75	0	52	29.9	42.8
23	50	64	57	55	100	100	96	28.5	67.2
24	75	100	25	55	100	100	44	47.6	46
26	0	100	40	20	100	0	40	42.9	32.9

The six patients with proximal femoral replacements who had the ability to walk had an average TESS of 72.5 % (SD ± 14.0; median 70.8; range 52.8–91.1), an average MSTS of 68.3 % (SD ± 25.5; median 68.4; range 33.3–96.7), and an average SF36 physical component summary score of 40.1 points (SD ± 11.2; median 42.9; range 24.9–53.7), whereas the average SF36 mental component summary score was 47.2 points (SD ± 15.0; median 45.9; range 29.7–67.2). The HHS of all patients with a functioning hip mega-prosthesis was 77.5 % on average (SD ± 17.7; median 81.0; range 51.0–94.0). The leg length measurement based on patient files or the functional follow-up assessment showed an average difference of −0.3 (±0.9 SD) cm (median 0.0; range −1.5 to 1.0) between the prosthesis leg and the healthy contralateral side.

## Discussion

### Aseptic loosening and nicotine consumption

The principal result of this study revealed that smoking is related to aseptic loosening in the mega-prosthetic replacement of the proximal femur. Thus, our hypothesis was valid for the present sample.

The aseptic loosening rate observed in this study (3/27) was comparable with the rates published in literature ranging from 5−27 % [1–3]. No other studies have identified smoking as a risk factor for aseptic loosening after the mega-prosthetic replacement of the proximal femur. However, the effects of smoking regarding the rates of aseptic loosening for other hip implants and bone healing are controversial. Some studies have not found a correlation between smoking and aseptic loosening after total hip arthroplasty [10, 11], whereas other studies have shown a correlation between smoking and aseptic loosening after total hip arthroplasty [12] as well as between smoking and revision total hip arthroplasty [13]. The latter observation is supported by our data.

Biochemically, several clues indicate how nicotine slows bone healing. Nicotine, through the activation of the cholinergic anti-inflammatory pathway, might partially and indirectly slow bone healing by inhibiting tumor necrosis factor-α (TNF-α) secretion [14]. TNF-α appears to be part of a cascade that leads to the expression of crucial proteolytic enzymes (matrix metalloproteinases 9, 13 and 14), which are necessary in fracture healing to progress from the soft callus stage to endochondral ossification [4, 15, 16]. Thus, the bone remains soft for a prolonged period in their absence. Delayed bone healing after the implantation of a proximal mega-prosthetic femoral replacement is extremely problematic because the femur and the implanted prosthesis must bear the patient’s full bodyweight during normal ambulation and stair climbing. During normal ambulation, this force can be 2.3 times the patient’s bodyweight [17]. Presumably, this delayed bone healing causes the unwanted prolonged motion of the implant within the bone and hinders proper osseointegration, thereby leading to aseptic loosening, even when the prosthesis is cemented.

In conclusion, smokers require special counseling before and after implantation of a mega-prosthetic replacement of the proximal femur to cease or at least reduce their smoking habits. They must be informed about their increased risk for aseptic loosening.

### Functional follow-up assessment: patient and prosthesis survival

The recent follow-up assessment revealed satisfactory results for all of the patients who survived with a functional hip prosthesis. This finding corroborates the current literature, which shows that successful functional results can be achieved for most patients after implantation of a mega-prosthetic replacement of the proximal femur [18, 19]. The Kaplan–Meier analysis showed that the cumulative survival of the prostheses was generally longer than the cumulative survival of the patients. In fact, the low patient survival rates were because of metastatic tumor diseases, which are the most common reasons for the implantation of these prostheses. After 7 years, we observed that prosthesis survival fell below patient survival. However, censoring of patients explains this change. Time will reveal how long the survivors will live with their prostheses and whether this effect will remain after additional years of observation.

### Limitations

One limitation of this study was clearly its small sample size. Because we included all of our patients with these types of prostheses over the past 10 years in this study, we were unable to increase our sample size. Although seven orthopedic surgeons performed this operation over the past decade, the surgical procedure, according to the study design, was the same for all of the patients and therefore provided comparable results. Apart from smoking, we were unable to identify any other factor that influenced aseptic loosening. Therefore, matching the smokers with comparable non-smokers based on sex, age, BMI, indication for operation, duration of operation, chemotherapy, radiation and implant at the surgical site prior to implantation would not have changed the results. However, we concede that matching was not possible because of the lack of appropriate matching partners. In addition, heterogeneous indications for implantation (malignant vs benign) and comorbidities led to strong differences regarding the survival rates of the patients.

## Electronic supplementary material

Below is the link to the electronic supplementary material.

Supplementary material 1 (DOCX 4 kb)
