# Supplementary material for: Nicotine consumption may lead to aseptic loosening in proximal mega-prosthetic femoral replacement
Source: J Orthop Traumatol. 2016 Aug 17;18(2):111–20. doi: 10.1007/s10195-016-0426-7 (PMC5429249; doi:10.1007/s10195-016-0426-7)
Supplement: Supplementary file 1 — Supplementary material 1 (DOCX 4 kb) [file 10195_2016_426_MOESM1_ESM.docx]

5-785.0f, 5-785.0g, 5-785.0h, 5-785.1f, 5-785.1g, 5-785.1h, 5-785.2f, 5-785.2g, 5-785.2h, 5-785.3f, 5-785.3g, 5-785.3h, 5-785.4f, 5-785.4g, 5-785.4h, 5-785.5f, 5-785.5g, 5-785.5h, 5-785.xf, 5-785.xg, 5-785.xh, 5-820.2, 5-820.20, 5-820.21, 5-820.22, 5-820.2y, 5-822.9, 5-822.90, 5-822.91, 5-822.92, 5-822.9y, 5-828.0, 5-828.1, 5-828.10, 5-828.2, 5-828.3, 5-828.4, 5-828.40, 5-828.5, 5-828.6, 5-829.c, 5-829.d, and 5-829.k.
